# A New Pre-descemetic Corneal Ring (Neoring) in Deep Anterior Lamellar Keratoplasty for Moderate-Advanced Keratoconus: A Pilot 2-Year Long-Term Follow-Up Study

**DOI:** 10.3389/fmed.2021.771365

**Published:** 2021-11-04

**Authors:** Belén Alfonso-Bartolozzi, Carlos Lisa, Luis Fernández-Vega-Cueto, David Madrid-Costa, José F. Alfonso

**Affiliations:** ^1^Fernández-Vega Ophthalmological Institute, Universidad de Oviedo, Oviedo, Spain; ^2^Faculty of Optics and Optometry, Universidad Complutense de Madrid, Madrid, Spain

**Keywords:** corneal transplant, deep anterior lamellar keratoplasty (DALK), visual outcomes, refractive outcomes, keratoconus

## Abstract

**Purpose:** To assess the outcomes of implanting a new polymethylmethacrylate (PMMA) ring (Neoring; AJL Ophthalmic) in pre-descemet deep anterior lamellar keratoplasty (PD-DALK) procedure for moderate-advanced keratoconus.

**Methods:** This prospective study included 10 eyes of 10 patients with moderate-advanced keratoconus who underwent PD-DALK with Neoring implantation. Neoring was implanted in a pre-descemetic pocket. The post-operative examination included refraction, corrected distance visual acuity (CDVA), corneal tomography, and endothelial cell density (ECD). The root mean squares (RMSs) for coma-like aberrations and spherical aberration were evaluated for a pupil size of 4.5 mm. The junctional graft (Tg) and host (Th) thicknesses were measured. The post-operative follow-up was 24 months.

**Results:** Post-operative CDVA was 0.82 ± 0.14 (decimal scale), 100% of the eyes achieved a CDVA of 0.7 (decimal scale). The refractive cylinder was −2.86 ± 1.65 2-years after surgery. No eyes had a post-operative refractive cylinder ≥5.00 D and in five eyes (50%), it was ≤2.50 D. At the last visit, the mean keratometry was 45.64 ± 1.96 D, the RMS for coma-like aberrations was 0.30 ± 0.15 μm and spherical aberration was 0.22 ± 0.09. The mean ECD remains without changes over the follow-up (*P* = 0.07). At the last visit, Tg and Th were 679.9 ± 39.0 and 634.8 ± 41.2 μm, respectively. The thickness of the complex (host-Neoring) was 740.6 ± 35.6 μm. In all cases, this thickness was thicker than Tg.

**Conclusion:** The results of this study suggest that PD-DALK along Neoring implantation is a viable, effective, and safe option to optimize the post-operative results for moderate-severe keratoconus.

## Introduction

Deep anterior lamellar keratoplasty (DALK) is a surgical procedure in which a corneal donor, free from endothelium and Descemet's membrane (DM), is transplanted in patients affected by a corneal disease, in which the endothelium is healthy and can be preserved. DALK may be classified into two types according to the technique used: with and without DM exposure, known as Descemet-DALK (D-DALK), and pre-descemet DALK (PD-DALK), respectively. Overall, DALK has significant advantages over penetrating keratoplasty (PKP), including reduced risk of endophthalmitis, no immune reaction, and minor loss of endothelial cell density (ECD), among others (1). However, DALK is not free of potential intra- and post-operative complications. Some of these complications are related to the corneal transplantation itself (complications associated with the graft-host interface, graft epithelial problems, graft or host cornea vascularization, suture-related complications, stromal rejection, post-operative refractive errors) (1–6). Others are more technique-dependent (D-DALK seems to provide a faster visual recovery than PD-DALK; however, D-DALK is associated with a higher risk of intraoperative complications) (7, 8).

The ocular disease leading to corneal transplantation might also influence the DALK-related complications (6, 9–11). Advanced keratoconus is the most common indication for corneal transplantation in many countries (12, 13). The most common intraoperative complication is the DM rupture (14), being mainly associated with the D-DALK technique (2, 7, 8, 15). Complications related to the graft-host interface and the irregularity of the recipient corneal bed may make it difficult to achieve an optimal visual restoration in patients with severe keratoconus (16, 17).

We designed a new ring made of polymethylmethacrylate (PMMA), named Neoring (AJL Ophthalmic, Spain), to overcome the potential complications of DALK in advanced keratoconus. First, the Neoring is conceived for the PD-DALK technique owing to the fact that it is implanted in a PD pocket created through the trephination periphery. As previously pointed out, the most common intraoperative complication (rupture DM) is mainly associated with D-DALK. Second, the Neoring implantation aims to regularize the graft-host interface and recipient corneal bed. Notedly, that corneal graft conforms, in part, to the recipient bed. Hence, the Neoring implantation might lead to a better corneal morphology restoration, consequently, improve the visual and refractive outcomes after surgery.

In the current prospective study, 10 eyes of 10 patients who underwent PD-DALK with Neoring implantation for advanced keratoconus were case per case analyzed over a follow-up of at least 24 months.

## Patients and Methods

This is a prospective case series study that included patients with moderate-advanced keratoconus, who underwent PD-DALK surgery with Neoring implantation at the Fernández-Vega Ophthalmological Institute in Oviedo, Spain. This study was conducted in compliance with the tenets of the Declaration of Helsinki. The study was approved by the Ethics Committee of the “Principado de Asturias” (Oviedo, Spain). After receiving a complete description of the nature of the study and the possible consequences of surgery, all patients provided informed consent.

Inclusion criteria were the presence of keratoconus, poor or unsatisfactory corrected distance visual acuity (CDVA) with spectacle ( ≤ 20/40), intolerance to contact lens, mean keratometry ≥50 D, corneal thickness in the area of the central 3 mm ≤450 μm, and axial length ≥23.50 mm. Exclusion criteria were an active ocular disease (other than keratoconus) and a history of ocular disorders with a potential impact on the variables under study (cataract, macular degeneration, glaucoma, retinal detachment, neuro-ophthalmic diseases, or ocular inflammation).

**Figure 1:**
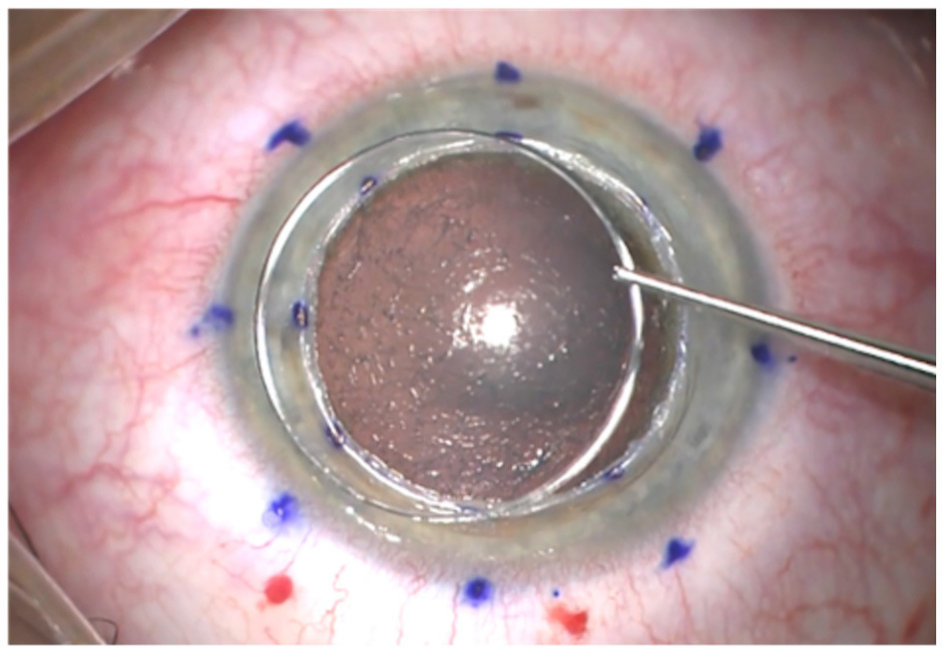
Image of Neoring before implantation.

All DALK surgeries were performed by the same surgeon (JFA) using Anwar's technique (18). Neoring (AJL Ophthalmic, Spain) was implanted in all cases. This ring is made of PMMA with a circular cross-section, a thickness of 0.21 mm, and it is available with three diameters (8, 8.5, and 9 mm). Neoring is conceived to be implanted in a pre-descemetic pocket created in the periphery of the trephination performed at the host cornea. The diameter of the Neoring must be 0.5 mm larger than the trephination diameter performed at the host cornea to achieve a correct ring fitting in the pre-descemetic pocket (Figure 1).

Briefly, the more significant steps of the surgical procedure were as follows: a partial trephination of the host corneal was performed with a diameter of 8 mm. The donor graft was cut 0.25 mm larger than the size used for the recipient corneal trephination. A manual layer-by-layer stromal dissection was performed, starting at the trephination edge down to the deepest stromal layer adjacent to DM. A thin stromal layer was left behind (between 50 and 70 μm at the central zone). A pre-descemetic pocket was created to 0.25 mm outside the trephination of the recipient cornea. Subsequently, a Neoring of 8.5 mm of diameter was implanted in the pre-descemetic pocket. The ring was provisionally fixated to the recipient cornea with two 8-0 silk sutures at 12 and 6 o'clock. Then, the donor button free from DM and endothelium was transferred to the recipient bed and sutured to the host corneal with four interrupted 10-0 nylon sutures (from 90% of the donor button depth and going the suture needle over Neoring to ensure that it is placed at pre-descemetic level). Finally, the silk sutures for the ring fixation were removed, and the 16 interrupted 10-0 nylon sutures were completed.

Post-operatively, selective suture removal started at 6 months after DALK, and it was completed at 15 months. The post-operative treatment consisted of a regimen of 1% dexamethasone and ciprofloxacin 0.3% drops four times a day for 1 week. Antibiotic drops were then discontinued, and dexamethasone was progressively tapered down over the next 3 months. Subsequently, we maintained dexamethasone in a regimen of one drop per day for a year. Furthermore, Plasma Rich in Growth Factors (PRGF) eye drops were added topically four times daily for at least 3 months.

**Figure 2:**
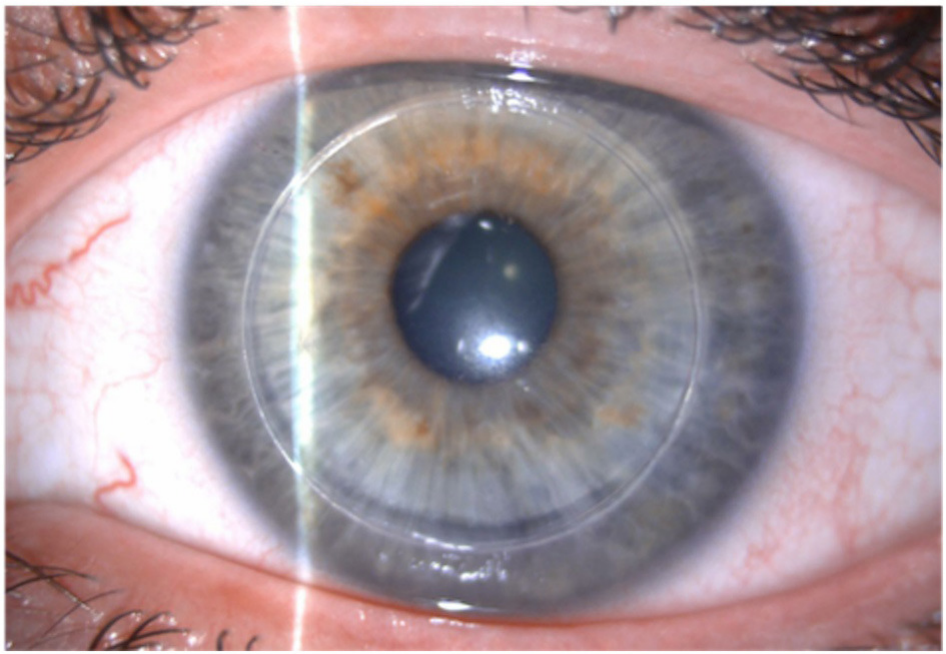
Neoring 24-months post-operatively.

Post-operative follow-up visits were scheduled 1-, 7-days, and 1 month after surgery and then every 3 months until 24 months (Figure 2). The post-operative examination included Manifest refraction, CDVA, slit lamp examination, corneal tomography (Sirius, CSO, Italy), ECD (SP 3000P, Topcon Europa), and corneal thickness (CCT) (Casia II- OCT, Tomey, Japan). Furthermore, at the last follow-up visit (24 months), the root mean square (RMS) for coma-like aberrations [computed for the Zernike terms Z (3, 1) and Z (3, −1)] and spherical aberration [Zernike coefficient Z (4, 0)] of the total cornea and the posterior corneal surface were evaluated for a pupil size of 4.5 mm. Finally, at the last visit, we used Casia II–OCT to measure the junctional graft thickness (Tg) and junctional host thickness (Th) and calculate the absolute value of the difference between Tg and Th (Tg-Th) according to the study of Zhao et al. (16). The corneal thickness of the complex host + Neoring was also measured.

Statistical analysis was carried out using Statistical Product and Service Solutions (SPSS) software for Windows (version 15.0, SPSS, Inc.). Pre-operative and post-operative data were compared using the Friedman test. A *P* < 0.05 was considered statistically significant. Data are reported as the mean ± SD.

## Results

The study included 10 eyes of 10 patients with a mean age of 36.90 ± 2.28 years (range 34–40 years). All patients completed the follow-up period of 2 years and attended all the follow-up visits. Table 1 summarizes the pre-operative data of the patients. All the surgeries were uneventful, with no intra or post-operative complications. No cases required conversion to PKP.

**Table 1:** Pre-operative data of the patients.

	**Mean ± SD**	**Range (Min, Max)**
Age (years)	36.90 ± 2.28	(34, 40)
Refraction sphere (D)	−7.80 ± 3.79	(−14.00, −3.00)
Refraction cylinder (D)	−8.40 ± 4.12	(−16.00, −4.00)
CDVA (Snellen Scale)	0.44 ± 0.07	(0.30, 0.50)
Axial length (mm)	25.45 ± 1.41	(23.51, 26.94)
Minimum keratometry (D)	51.85 ± 3.41	(48.12, 57.56)
Maximum keratometry (D)	59.55 ± 5.20	(53.18, 66.75)
Mean keratometry (D)	55.70 ± 3.98	(50.65, 61.50)
Corneal thickness (μm)	390 ± 52	(299, 444)
ECD (cells/mm^2^)	2,450 ± 543	(1,674, 3,522)

*D, diopters; CDVA, corrected distance visual acuity; ECD, endothelial cell density; SD, standard deviation*.

**Figure 3:**
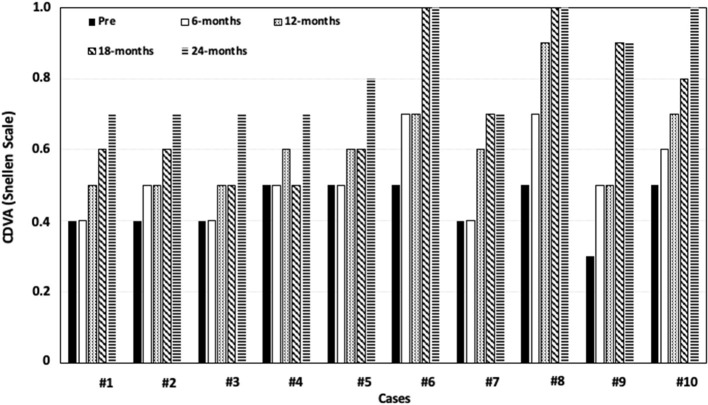
Variation in corrected distance visual acuity (CDVA) over the whole follow-up for each case.

Table 2 shows the pre-operative and post-operative clinical outcomes. All visual and refractive parameters significantly improved after DALK. CDVA was progressively improving visit by visit in all eyes until 24 months of follow-up. Figure 3 shows the changes in CDVA for each case studied over the whole follow-up. At the last visit, 100% of the eyes achieved a CDVA of 0.7 (decimal scale) and 50% of the eyes achieved a CDVA of 0.8 (decimal scale) or better. The safety index (ratio between the post-operative CDVA and the pre-operative CDVA) was 1.87.

**Table 2:** Pre-operative and post-operative clinical outcomes.

	**Pre-operative**	6-month^*^	**12-month**	18-month^**^	**24-month**
Refraction sphere (D) Range	−7.80 ± 3.79 (−14.00 to −3.00)	1.90 ± 3.90^#^ (−9.00 to +4.00)	−1.75 ± 2.20 (−5.00 to +1.00)	−3.03 ± 2.42^#^ (−7.00 to 0.00)	−3.39 ± 2.58 (−8.00 to −0.50)
Refraction cylinder (D) Range	−8.40 ± 4.12 (−16.00 to −4.00)	−3.40 ± 1.71^#^ (−6.00 to −0.50)	−3.65 ± 1.80 (−6.00 to −1.50)	−2.75 ± 2.01^#^ (−5.50 to −0.25)	−2.86 ± 1.65 (−5.00 to −1.00)
CDVA (Snellen scale) Range	0.44 ± 0.07 (0.30 to 0.50)	0.52 ± 0.11^#^ (0.40 to 0.70)	0.61 ± 0.13^#^ (0.50 to 0.90)	0.72 ± 0.19^#^ (0.50 to 1.0)	0.82 ± 0.14^#^ (0.70 to 1.0)
Keratometric cylinder (D) Range	7.70 ± 3.73 (2.25 to 14.49)	4.09 ± 2.92^#^ (0.49 to 8.39)	3.56 ± 1.44 (2.08 to 5.59)	3.12 ± 1.43 (1.04 to 4.82)	3.19 ± 1.26 (1.04 to 4.87)
Minimum keratometry (D) Range	51.85 ± 3.41 (48.12 to 57.56)	42.19 ± 4.00^#^ (35.62 to 48.78)	43.14 ± 2.88 (38.66 to 47.91)	43.88 ± 2.36 (39.83 to 47.21)	44.05 ± 1.89 (42.13 to 47.21)
Maximum keratometry (D) Range	59.55 ± 5.20 (53.18 to 66.75)	46.27 ± 3.13^#^ (42.41 to 51.34)	46.70 ± 2.01 (44.18 to 50.88)	47.00 ± 2.40 (44.23 to 52.03)	47.22 ± 2.19 (44.80 to 51.92)
Mean keratometry (D) Range	55.70 ± 3.98 (50.65 to 61.50)	44.23 ± 3.28^#^ (39.02 to 50.06)	44.92 ± 2.37 (41.46 to 49.40)	45.44 ± 2.27 (42.01 to 49.62)	45.64 ± 1.94 (43.47 to 49.57)
Corneal thickness (μm) Range	389.50 ± 51.58 (294 to 444)	541.70 ± 34.60^#^ (502 to 595)	550.50 ± 30.17 (501 to 589)	554.56 ± 21.89 (510 to 580)	566.56 ± 30.65 (520 to 620)
ECD (cells/mm^2^) Range	2,450 ± 543 (1,674 to 3,522)	2,396 ± 399 (1,874 to 3,157)	2,498 ± 277 (2,162 to 3,101)	2,591 ± 444 (2,065 to 3,210)	2,584 ± 283 (2,185 to 3,104)

* *Starting of sutures removal (16 interrupted 10-0 nylon sutures in place);*

** *3 months after completed sutures removal;*

# *Statistically significant differences with the previous follow-up visit; D, diopters; CDVA, Corrected Distance Visual Acuity; ECD, endothelial cell density*.

**Figure 4:**
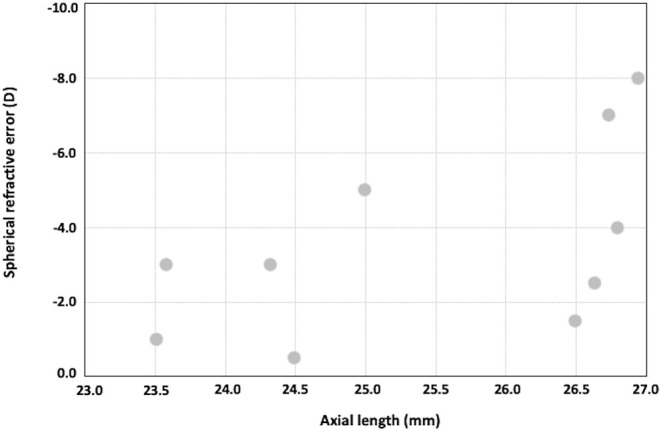
Plots showing axial length vs. post-operative spherical refractive error.

The spherical component of the refraction dropped from a pre-operative −7.80 ± 3.79 D to a 24-month post-operative value of −3.39 ± 2.58 D (*P* = 0.0001). At the last visit, only four eyes had a spherical refractive error ≥4.00 D. In these cases, the axial length was longer than 25 mm (Figure 4). The refractive cylinder changed from −8.40 ± 4.12 D pre-operatively to −2.86 ± 1.65 D 2 years after surgery (*P* < 0.001). At the last visit, no eyes had a post-operative refractive cylinder ≥5 D, and in five eyes (50%), it was ≤2.50 D. Both, the maximum and minimum K reading values, declined significantly after surgery. At the last visit, the mean keratometry was 45.64 ± 1.94 D (ranging from 43.47 to 49.57 D).

Regarding corneal aberrometry, the 24-month post-operative RMSs for coma-like and spherical aberrations of the posterior corneal surface were 0.12 ± 0.09 and 0.08 ± 0.18 μm, respectively. In turn, the RMS for coma-like aberrations of the total corneal was 0.30 ± 0.15 μm and the spherical aberration was 0.22 ± 0.09 μm.

There were no significant changes in the mean ECD at any timepoint (*P* = 0.07). The mean CCT increased from 389.4 ± 51.58 μm pre-operatively to 541.7 ± 34.6 μm at 6 months post-operatively (*P* = 0.0006), and then it remains unchanged over the follow-up (566.56 ± 30.65 μm at the last visit).

**Figure 5:**
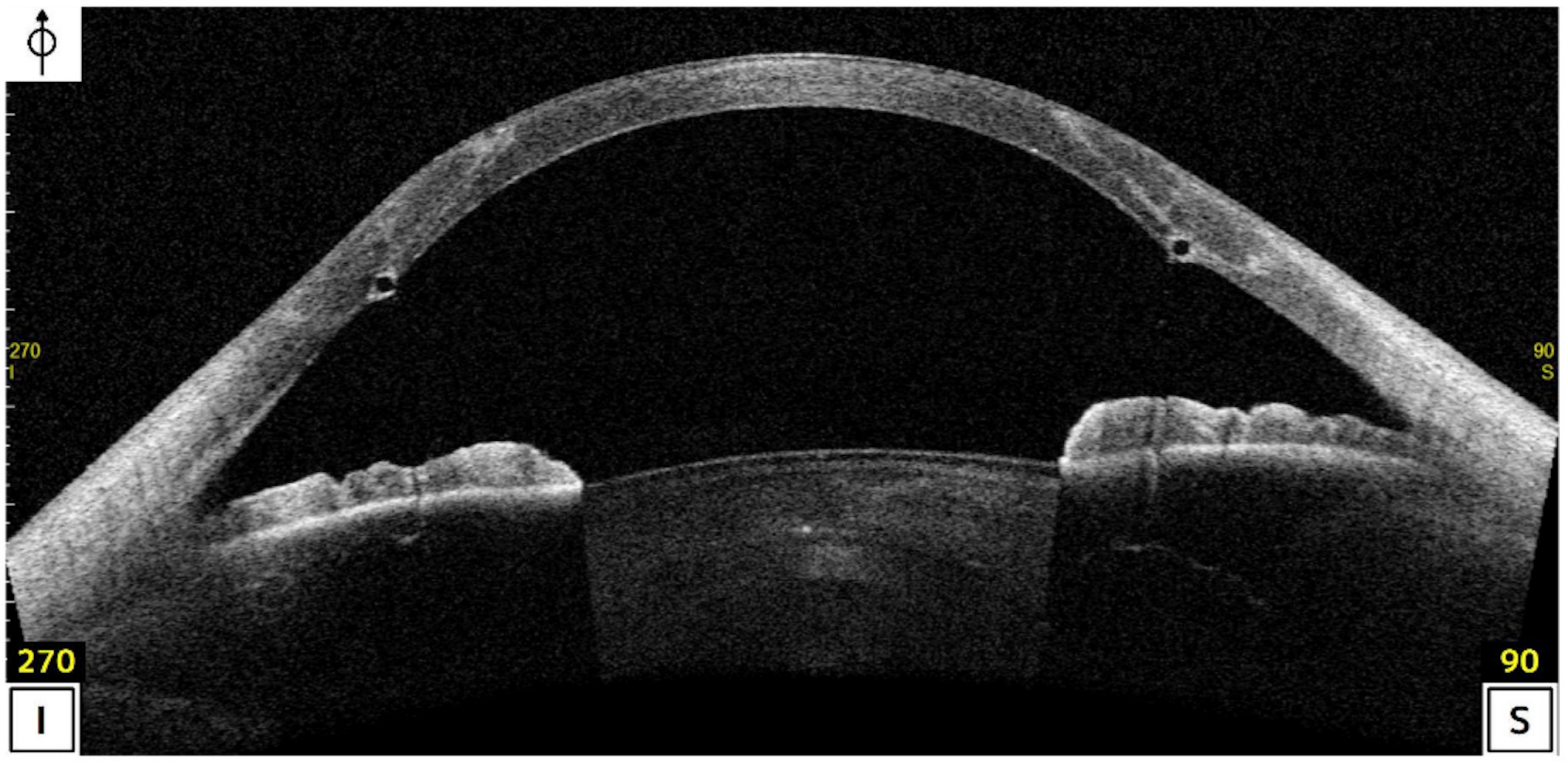
Anterior Segment Optical Coherence Tomography (AS-OCT; Casia II- OCT, Tomey, Japan) 24-months post-operatively.

At the last visit, Tg and Th were 679.9 ± 39.0 and 634.8 ± 41.2 μm, respectively. Consequently, the (Tg-Th) was 45.1 ± 24.02 μm. The thickness of the complex host + Neoring was 740.6 ± 35.6 μm, and in all cases, this thickness was thicker than Tg (Figure 5).

## Discussions

Advanced keratoconus is a corneal disease that causes severe visual impairment and is a leading indication for corneal transplantation (12, 13). Notedly, keratoconus, even in its most advanced stages, affects young people; consequently, achieving a good visual quality that lasts for a lifetime must be the main aim.

DALK in keratoconus aims to improve visual quality through the restoration of corneal morphology. The graft-host matching is a key factor to achieve optimal visual and refractive outcomes (16). The thickness of the corneal bed is also a factor that limits the visual acuity restoration after DALK, a recipient thickness of more than 80 μm reduces the post-operative visual acuity (17). In turn, the posterior corneal surface is also affected by keratoconus (19). It should be kept in mind that the graft conforms, at least in part, to the recipient DM, which remains intact after DALK. Therefore, the changes and the irregularities induced by keratoconus in the posterior corneal surface could contribute to worsening the optical quality of the corneal graft.

This pilot study evaluated the clinical outcomes of implanting a new PMMA ring (Neoring) in a pre-descemetic pocket in the PD-DALK procedure. PD-DALK for keratoconus may provide comparable CDVA outcomes to D-DALK (2, 7, 8, 20, 21), as long as, as noted above, the recipient bed thickness is thinner than 80 μm (17). Consequently, in our study, the stromal layer left behind was thinner than 70 μm at the central zone in all cases. Furthermore, the Neoring implantation aimed to achieve a better corneal morphology restoration by increasing the thickness of the retained peripheral recipient cornea and regularizing the recipient corneal bed.

The results showed satisfactory visual acuity. At the last visit, the CDVA was 0.82 ± 0.14 (decimal scale) (range from 0.7 to 1.0), 100% of the eyes achieved a CDVA of 0.7 (decimal scale), and 50% of the eyes achieved a CDVA of 0.8 (decimal scale) or better. Furthermore, it is important to note that three of the five eyes with a CDVA of 0.7 (decimal scale) had an axial length longer than 26.5 mm and a posterior staphyloma that reached the macular area. Hence, it seems that likely these cases obtained their maximum potential visual acuity. The safety index ratio was 1.87. Our visual results were better than those previously reported in the more advanced keratoconus. In the study of Feizi et al. (11), the CDVA was 0.19 and 0.20 logMAR (around 0.6 in decimal scale) for stage III and IV, respectively. Javadi et al. (22), in turn, reported a CDVA of 0.16 and 0.18 logMAR (about 0.7 decimal scale) for moderate and severe keratoconus using D-DALK. This study reported significantly worse CDVA for severe keratoconus with the PD-DALK technique (0.29 logMAR; around 0.5 in decimal scale). We should be cautious with these comparisons because although our cases were prospectively analyzed, this is a pilot study including 10 eyes. However, it seems plausible that the Neoring implantation might benefit visual acuity restoration, as will be argued below.

Regarding refractive outcomes, at the last visit, only four eyes had a spherical refractive error ≥ 4 D. In these cases, the axial length was longer than 25 mm (Figure 4). Furthermore, the mean keratometry at the last visit was 45.64 ± 1.94 D. These results indicate that the post-operative sphere refraction was mainly associated with the elongation of the posterior segment, and not with a significant steepness of the corneal graft. The refractive cylinder changed from −8.40 ± 4.12 D pre-operatively to −2.86 ± 1.65 D 2 years after surgery (*P* < 0.001). The average reported refractive cylinder after DALK for keratoconus may vary among studies between 2.50 and 5.00 D (2, 23, 24). For the moderate and severe keratoconus, the reported post-DALK astigmatism is around 3.50 D but may reach up to 10.00 D (11). In the present study, at the last visit, no eyes had a post-operative refractive cylinder ≥5.00 D and in five eyes (50%), it was ≤ 2.50 D.

According to the visual and refractive outcomes found in this pilot study, it seems that PD-DALK along Neoring implantation might represent a viable option to optimize the post-operative results in moderate-severe keratoconus. We have reasons to accept the assumption that Neoring implantation influenced these results. First, it should be noted that the donor graft conforms, at least in part, to the morphology of the recipient intact bed. Therefore, it would imply that the more irregular the recipient bed, the worse regain the corneal refractive regularity. Advanced keratoconus has steeper corneas, and the posterior cornea is more irregular. Hence, it should be expected that an action to regularize the recipient bed might improve the post-operative visual and refractive outcomes. We hypothesized that a healing process mediated by activated keratocytes might occur around Neoring. In a similar way to what happens after intracorneal ring segment implantation (25). This wound-healing process might apply a pushing force on the periphery, which might lead to a flattening of the recipient corneal bed. Consequently, and considering that the recipient corneal radii of curvature are a factor influencing the post-operative graft radii of curvature (26), it might result in a less steep keratometry of the corneal graft. The post-operative mean keratometry in our study was 45.64 ± 1.94 D with no cases exceeded 50 D. In contrast, the post-operative mean keratometry reported by Feizi et al. (11) in stages III and IV were around 1.0 and 1.5 D stepper, respectively, and some cases had a Km up to 53.50 and 56.50 D, respectively.

Furthermore, Neoring implantation might mitigate the irregularities of the recipient bed induced by advanced keratoconus, and as a consequence, the total corneal aberrations. The 24-month post-operative RMSs for coma-like and spherical aberrations of the posterior corneal surface were 0.12 ± 0.09 and 0.08 ± 0.18 μm, respectively. In turn, the RMS for coma-like aberrations of the total corneal was 0.30 ± 0.15 μm and the spherical aberration was 0.22 ± 0.09 μm. We cannot compare it with the pre-operative values. However, considering that posterior corneal aberrations increase in advanced keratoconus, it should be expected that the posterior coma-like and spherical aberrations were significantly higher before surgery. Besides, our research group has recently shown that long-arc-length intrastromal corneal ring (ICRS) implantation provides significant changes in the anterior and posterior corneal surface morphology of moderate-advanced keratoconus, becoming both surfaces more regular (27). Although both, ICRS and Neoring, have different indications and mechanisms of action, it seems plausible to expect that Neoring might also have a regularization effect on the recipient bed. Moreover, the continuous improvement in CDVA visit by visit (Table 2) could be related, obviously, to the removal of the sutures and an ongoing regularization process that, in turn, would be influenced by the healing process around the ring previously explained.

Finally, it is essential to note that the implant added volume to the retained peripheral recipient cornea (Figure 5), decreasing the difference between the Tg and Th (Tg-Th). Zhao et al. (16) found that the larger the (Tg-Th) value, the worse the visual and refractive outcomes. The (Tg-Th) value was lower in our study than that reported by Zhao et al. (16) (45.1 and 85.74 μm, respectively). Furthermore, the thickness of the complex (host + Neoring) was thicker than Tg in all cases. All these findings suggest that Neoring implantation in PD-DALK would provide that the posterior corneal surface became more regularized and flattened post-operatively and a smoother transition graft-host junction, providing encouraging outcomes in advanced keratoconus.

In the current study, no complications occurred during surgery or over the entire follow-up time. The most common and significant intraoperative complication is the DM perforation, being mainly associated with the D-DALK technique (2, 7, 8, 15). The endothelial cell count remained stable over the follow-up, suggesting that Neoring implantation did not cause alterations in the endothelium. A late post-operative complication after DALK is the recurrent keratoconus (2, 28, 29). The difference between Tg and Th might provoke a progressive thinning of the graft-host junction over time, which might induce high irregular astigmatism. As previously explained, the implant added volume to the retained peripheral recipient cornea (Figure 5), decreasing the difference between Tg and Th, and making the thickness of the complex (host + Neoring) thicker than Tg. Furthermore, as Krumeich proposed (30), ring implantation might secure the stability of the wound. Hence, the approach of implanting Neoring might minimize the risk for recurrent keratoconus. However, further studies with a larger sample and longer follow-ups are required to address these potential benefits.

Despite the encouraging results in the prospective study, it should be pointed out that this is a pilot study including 10 cases and it only represented a single-center experience. It is important also to note that all surgeries were carried out by an experienced surgeon. Therefore, further prospective, randomized, and multicenter studies with a larger sample and longer follow-ups are required to deeply address the potential benefits of this procedure. Notedly, Neoring was initially conceived for the PD-DALK technique because it is implanted in a PD pocket. However, it should be noted that femtosecond laser can be safely and effectively used to create cutting patterns (31) in the host and donor corneas that might allow Neoring implantation in a PD pocket in the periphery, leaving bared a central region of Descemet's membrane. Hence, we consider it interesting to evaluate the outcomes of Neoring implantation in femtosecond laser-assisted DALK using those cutting patterns (31) in future studies.

In conclusion, the findings of this pilot study suggest that Neoring implantation in the PD-DALK procedure might represent a viable option to optimize the post-operative results in moderate-severe keratoconus. Further, long-term follow-up studies, including a larger number of cases, are needed to properly analyze the stability of this surgery and confirm the safety of the procedure.

## Data Availability Statement

The original contributions presented in the study are included in the article, further inquiries can be directed to the corresponding author.

## Ethics Statement

The study was approved by the Ethics Committee of the Principado de Asturias (Oviedo, Spain). The patients/participants provided their written informed consent to participate in this study.

## Author Contributions

BA-B: conception and design of the study, analysis and interpretation of data, writing the manuscript, and critical revision of the manuscript. CL and LF-V-C: conception and design of the study, analysis and interpretation of data, and critical revision of the manuscript. DM-C and JA: conception and design of the study, analysis and interpretation of data, critical revision of the manuscript, and supervision. All authors read and approved the final manuscript.

## Funding

Fernández-Vega Ophthalmological Institute has an unrestricted Grant from AJL Ophthalmic.

## Conflict of Interest

The authors declare that the research was conducted in the absence of any commercial or financial relationships that could be construed as a potential conflict of interest.

## Publisher's Note

All claims expressed in this article are solely those of the authors and do not necessarily represent those of their affiliated organizations, or those of the publisher, the editors and the reviewers. Any product that may be evaluated in this article, or claim that may be made by its manufacturer, is not guaranteed or endorsed by the publisher.
